# Cross-domain processing of musical and vocal emotions in cochlear implant users

**DOI:** 10.3389/fnins.2015.00343

**Published:** 2015-09-24

**Authors:** Alexandre Lehmann, Sébastien Paquette

**Affiliations:** ^1^Department of Otolaryngology Head and Neck Surgery, McGill UniversityMontreal, QC, Canada; ^2^International Laboratory for Brain, Music and Sound Research, Center for Research on Brain, Language and MusicMontreal, QC, Canada; ^3^Department of Psychology, University of MontrealMontreal, QC, Canada

**Keywords:** cross-domain processing, emotion, music, voice, cochlear implant, brain plasticity, neural overlap

Music and voice bear many similarities and share neural resources to some extent. Experience dependent plasticity provides a window into the neural overlap between these two domains. Here, we suggest that research on auditory deprived individuals whose hearing has been bionically restored offers a unique insight into the functional and structural overlap between music and voice. Studying how basic emotions (happiness, sadness, and fear) are perceived in auditory stimuli constitutes a favorable terrain for such an endeavor. We outline a possible neuro-behavioral approach to study the effect of plasticity on cross-domain processing of musical and vocal emotions, using cochlear implant users as a model of reversible sensory deprivation and comparing them to normal-hearing individuals. We discuss the implications of such developments on the current understanding of cross-domain neural overlap.

## Cross-domain neural overlap and plasticity

Our musical and vocal perception abilities have such a close relationship that some authors suggested that the former originated from the latter or vice-versa (Honing et al., 2015; Peretz et al., 2015). To what extent do music and voice share functional and structural networks and at which stage of auditory processing they are differentiated are open questions. Functional magnetic resonance imaging (fMRI) studies show the co-activation of brain regions with possibly distinct underlying neural populations (Peretz et al., 2015). Research on expert populations has suggested reciprocal interactions between neural circuits associated with the domains of music and voice (Patel, 2011; White-Schwoch et al., 2013; summarized by Paquette and Mignault Goulet, 2014). Indeed studies have shown that musicians have enhanced speech processing capacity, which is reflected in both cortical and subcortical neural measures (Bidelman et al., 2011, 2014; Parbery-Clark et al., 2012). Musicians can be used as a model of learning-induced plasticity to investigate how such cross-domain transfer effects unfold over time (Strait and Kraus, 2014; Strait et al., 2014). Here we argue that, sensory deprivation offers a complementary model to shed light on the plastic reorganization of brain networks involved in particular functions.

## Temporary deafened individuals offer a unique insight into auditory neural plasticity

Cochlear implants (CI) are bionic devices that can restore the sense of hearing in profoundly deaf individuals. We argue that cochlear implant users offer a promising model to study the mechanisms of cross-domain plasticity because they undergo different trajectories of auditory development: deafness of various origins results in a variable period of auditory deprivation followed by surgical restoration of auditory input and an intense rehabilitation period, yielding variable individual auditory outcomes.

Signal transmitted from the implant to the auditory nerve is impoverished compared to natural hearing. Critically, the access to pitch cues is impaired, reduced to a small number of frequency bands. As a result, cochlear implant users can potentially perceive speech relatively well in a quiet setting, but understanding it in noise, or accurately perceiving music is very challenging since both tasks rely on pitch information (Gfeller et al., 2007). Perception is not only affected by the impoverished auditory input, but also by neural re-organization following auditory deprivation, from the periphery to the cortex. In absence of auditory input, auditory nerve fibers start to degenerate and the auditory cortex can be recruited by visual and somatosensory systems (Collignon et al., 2011; Lazzouni and Lepore, 2014). Such plastic changes can prevent the auditory cortex from fully recovering its initial function after the auditory input is restored via an implant (Lee et al., 2001; Bavelier and Hirshorn, 2010; Sandmann et al., 2012; Sharma et al., 2015).

To date, little is known of the neural correlates of music and voice processing in cochlear implants and the extent to which those processes overlap. Only one study has performed a direct comparison of the neural correlates of speech and music perception in CI users. Using positron emission tomography (PET), Limb et al. (2009), reported increased activation and greater cortical recruitment in implant recipients compared to normal hearing controls, during both speech and music listening. This effect was stronger for speech—for which CI users are more proficient than music—and suggest a link between auditory performance and degree of auditory cortical activation.

## Emotion as a cross-domain terrain of choice to study neural overlap

An important part of our social interaction relies on accurate emotion perception. In normal-hearing individuals, evidence from neuropsychology suggest the existence of an auditory emotional neural pathway, distinct from auditory perception, that might be shared across musical and vocal domains and have both cortical and subcortical components (Peretz, 2011). A systematic comparison of the vocal and musical domains suggests a close acoustical relationship for emotional expression, with similar emotion-specific acoustic cues patterns (Juslin and Laukka, 2003). Several of those patterns relate to the pitch dimension, such as prosody for voice (variations in the pitch contour) and melody for music. The perception of pitch is severely degraded in cochlear implant users, thus limiting their access to those important cues, but other non-pitch based cues can also convey emotions (Gabrielsson and Lindström, 2010). It was recently demonstrated in amusics (individuals with a lifelong pitch perception deficit; Peretz, 2013) that non-pitch based cues (e.g., tempo, pulse clarity) can be used to identify musical emotions (Gosselin et al., 2015). These cues are available to some extent to CI users (Kong et al., 2004; Looi et al., 2012), and should allow them a certain degree of emotional perception. CI users have a documented deficit in both vocal and musical emotion recognition; emotional categories and dimensions are not uniformly impaired. They can recognize some categories of emotion in voice or music above chance, but not as well as normal hearing controls (Hopyan et al., 2012; Nakata et al., 2012; Volkova et al., 2013; Wang et al., 2013). They have difficulty perceiving arousal of musical excerpts but not valence (Ambert-Dahan et al., 2015). These differences could be due to the relatively spared abilities of CI users to perceive temporal variations, while having an impaired pitch perception. They could also reflect differences in the complexity of stimuli employed and how they are handled by speech-optimized processors, suggesting that *ad-hoc* stimuli are required to accurately compare the two domains. This could explain why no study has yet directly compared emotion processing in CI users across the domains of music and voice. To date, there is very little neuro-imaging evidence building up on the aforementioned behavioral findings. Only one study evaluated the impact of two implant processing strategies on the perception of prosody (Agrawal et al., 2013) and demonstrated that electroencephalography (EEG) is a useful tool to reveal differences between strategies coding specific features.

## Toward a study of cross-domain processing of musical and vocal emotions in cochlear implant users

A large part of the research on auditory affective processing has been conducted on prosody utilizing words or sentences spoken with various emotional expressions and complex musical pieces expressing varying degrees of emotion. It is not possible to directly compare those results between music and voice because of many confounding variables; factors such as speech semantics, length, harmony, and context are likely to recruit different neural networks. We argue that a necessary first step to study cross-domain processing of musical and vocal emotions is to use an experimental paradigm that moves away from the fairly complex sounds used in the existing literature, using stimuli that enable a controlled comparison between the domains of music and voice. A possible approach would be to use the most primitive affect expressions (primal interjections close to those of babies and animals) in each domain: non-speech vocalizations and brief mono-instrumental musical excerpts.

In the vocal domain, non-speech vocalizations (e.g., screams, laughter) depicting basic emotions that are minimally conventionalized, relatively universal and fundamental to spontaneous human communication (Scherer, 1986), could be used. Stimuli like the Montreal Affective Voices (Belin et al., 2008), consisting of short vocal interjections on the vowel /a/ expressing basic emotions, represent the most primitive form of emotion in their domain. They have minimal semantic information and minimal interaction with linguistic processes (Bestelmeyer et al., 2010). Compared to speech prosody, vocalizations are treated preferentially in the brain (Pell et al., 2015). When it comes to music, finding the most basic emotions and avoiding interaction with other processes require stepping away from conventional structure (limited by mode or tempo), reducing the length of the stimuli and reducing its emotional complexity. Stimuli like the Musical Emotional Bursts (Paquette et al., 2013) could be used for comparison, they consist of a few spontaneous notes on a clarinet or violin expressing basic musical emotions, they are minimally conventionalized and represent the most primitive form of emotion in their domain. They are all the more similar to vocal stimuli because they use continuous pitch instrument (e.g., the violin which offers a seamless progression between notes, giving the stimuli a quasi-vocal quality), whereas most studies have used discrete pitch instruments (e.g., the piano where one key corresponds to one pitch), which further hinders the direct comparison with vocal stimuli.

These highly similar vocal and musical stimuli seem well-suited to study cross-domain overlap in any population and their primitive quality could be extremely useful to study plasticity in CI users.

A second step would be to pair a well-controlled behavioral paradigm using those stimuli (allowing a direct comparison of musical and vocal domains) with a neuro-imaging modality that is acceptable for use with cochlear implants. Except for a few recent exceptions, implants are not MR-compatible. Hi-density EEG (Gilley et al., 2010; Zhang et al., 2011; Timm et al., 2014) and PET-scan (Okazawa et al., 1996; Limb et al., 2009; Lazard et al., 2010) have both been used successfully in cochlear implant users. Both methods have drawbacks; EEG recordings are contaminated by massive electrical artifacts from the implant and PET requires the injection of a radioactive isotope. Emerging as a promising brain-imaging modality for CI research is functional near-infrared spectroscopy (fNIRS). fNIRS has been successfully used to study the response to auditory stimuli in cochlear implant users (Sevy et al., 2010) and emotion-related activation in the general population (Herrmann et al., 2003; Plichta et al., 2011). This non-invasive technique measures blood oxygenation level differences using infrared light and is therefore unaffected by electrical artifacts. It is portable and has a better temporal resolution than functional MRI (Villringer and Chance, 1997). Conversely it has a worse spatial resolution and cannot access subcortical sources such as the limbic system (Köchel et al., 2011).

The proposed neuro-behavioral approach would be well-suited to study the effect of plasticity on cross-domain processing of musical and vocal emotions, using cochlear implant users as a model of reversible sensory deprivation and comparing them to normal-hearing individuals. The effect of multiple regressors could be assessed by recruiting an heterogeneous cohort of individuals spanning the continuum of factors known to affect plasticity such as the duration of auditory deprivation or the age at implantation (Lazard et al., 2012).

This would represent a stepping-stone to ask further questions of interest regarding the effect of plasticity on cross-domain neural overlap. From a basic science perspective, the rationale is to understand a complex system by reverse-engineering its dysfunctions. What are the structural and functional overlaps between music and voice processing after implantation? Would the reduction of auditory cortical resources, together with the fact that music and vocal signals are more similar after being processed by the device, favor an increased neural overlap between domains? Conversely, would any remaining overlap break-down in favor of a more segregated re-organization guided by the non-pitch based, domain relevant cues?

Characterizing those mechanisms can inform novel clinical approaches, possibly through individualized rehabilitation and brain stimulation. For instance, if good performers (CI users with good speech scores) make use of overlapping structures in an optimal fashion compared to poor performers, can we boost residual neural processes in the latter group? It has been suggested that musical training can improve speech outcomes in this population (Patel, 2014), but what stages of the auditory pathway are best candidates for a cross-domain shaping of function and/or structure? Auditory features found to maximize activity of brain networks processing musical and vocal emotions in CI users could be made more salient in device processors.

Cross-domain research on cochlear implant users not only offers a unique insight into auditory neural plasticity, but also has practical implications for patients' rehabilitation, implant design, and programming. We believe that highly comparable stimuli are needed to carry out such studies, together with an optimal imaging technique within a paradigm fine enough to reveal subtle behavioral and neural differences. Such scientific undertaking can further our understanding of how our brain processes vocal and musical emotions and how such cross-domain processing is affected by plasticity. Furthermore, such studies could provide objective measures to support the use of music in the rehabilitation of various disorders.

### Conflict of interest statement

The authors declare that the research was conducted in the absence of any commercial or financial relationships that could be construed as a potential conflict of interest.
